# The intersegmental pulmonary vein is not always located on the intersegmental plane of the lung: Evaluation with 3-dimensional volume-rendering image reconstruction

**DOI:** 10.1016/j.xjtc.2022.09.002

**Published:** 2022-09-13

**Authors:** Takahiro Mimae, Yoshihiro Miyata, Takashi Kumada, Yasuhiro Tsutani, Morihito Okada

**Affiliations:** Department of Surgical Oncology, Hiroshima University, Hiroshima, Japan

**Keywords:** intersegmental pulmonary vein, segmentectomy, blood flow, reconstruction, 3D, three-dimensional, CT, computed tomography, HU, Hounsfield unit

## Abstract

**Objective:**

To clarify whether intersegmental pulmonary veins are always located on the intersegmental plane and determine the division from which blood flows into them.

**Methods:**

We analyzed representative intersegmental veins located between the upper/lingular and superior/basal division of the lungs using preoperative chest computed tomography (CT) DICOM data from 22 patients who underwent lobectomy or segmentectomy during 2020. The location and blood flow of V^3^a+b and V^6^b+c were assessed using REVORAS (Ziosoft), a novel volume-rendering 3-dimensional (3D) image reconstruction software dedicated to lung segmentectomy.

**Results:**

The V^3^a+b was in the upper division and on the intersegmental plane between the upper and lingular divisions of the left lung in 11 patients (50%) each. A main root of V^3^a+b was not found in the lingular division, but some peripheral flow in the V^3^a+b was derived from it in 14 patients (64%). The V^6^b+c was found in the superior division of the right lower lobe in 13 patients (59%) and the left lower lobe in 10 patients (45%), and on the intersegmental plane between the superior and basal division of the right lower lobe in 6 patients (27%) and the left lower lobe in 10 patients (45%). A main root of V^6^b+c was imperceptible in the basal division. Some peripheral blood flow was derived from the basal division in 6 patients (27%) with V^6^b+c veins located in the right lower lobe and in 8 patients (36%) with V^6^b+c veins located in the left lower lobe.

**Conclusions:**

Precise evaluation of intersegmental veins using preoperative volume-rendering 3D reconstructed CT images provides useful anatomic information for separating intersegmental pulmonary parenchyma.

## Graphical abstract

**Figure:**
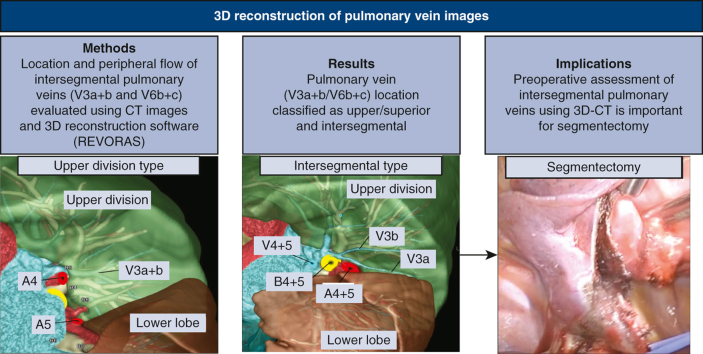

**Figure:**
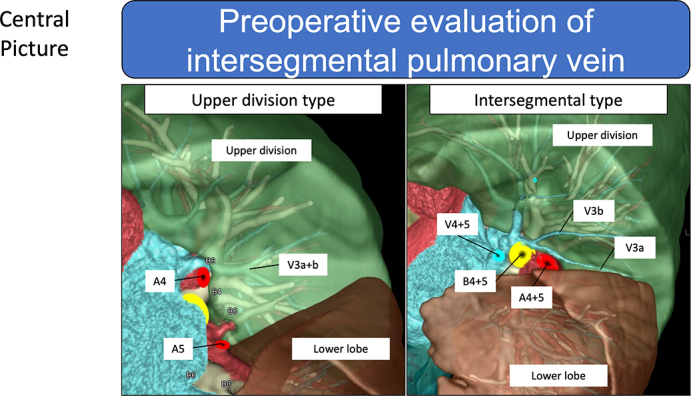
Intersegmental vein V^3^a+b is classified into upper and intersegmental types.

**Central Message**

Evaluation of intersegmental veins using preoperative 3-dimensional reconstruction of computed tomography images is useful for separating intersegmental pulmonary parenchyma in segmentectomy.

**Perspective**

This study revealed the location of intersegmental pulmonary veins (V^3^a+b and V^6^b+c) and the source and direction of blood flow using preoperative 3-dimensional reconstruction of computed tomography images. This information is important for separating intersegmental pulmonary parenchyma at the dawn of a new era in segmentectomy. Our findings require further validation in different cohorts.

Segmentectomy is a key surgical procedure that general thoracic surgeons will need to master soon.^1^^,^^2^ The Japanese Clinical Oncology Group 0802 (JCOG0802)/West Japan Oncology Group 4607L (WJOG4607L) prospective randomized trial found significantly better overall survival after segmentectomy than after standard lobectomy with mediastinal lymph node dissection.^3^ Thus, segmentectomy will soon become the standard surgical procedure for small and peripherally located non–small cell lung cancer, as well as a passive indication for compromised patients.

Intersegmental veins are key anatomic structures in terms of segmentectomy owing to their landmark of separating intersegmental parenchyma. Therefore, understanding their precise anatomic location is essential for good outcomes of lung surgery. Preoperative three-dimensional (3D) reconstructed anatomic structures associated with pulmonary resection based on computed tomography (CT) images are useful for lung resection, as well as for safe and reliable anatomic resections, lobectomy, and segmentectomy.^4^, ^5^, ^6^, ^7^, ^8^, ^9^, ^10^, ^11^, ^12^ However, the precise location and flow of intersegmental pulmonary veins has not been defined—that is, whether the representative intersegmental pulmonary vein V^3^a+b between the upper (S^1-3^) and lingular (S^4+5^) divisions of the left lung is always located on the intersegmental plane or can be located closer to the upper/lingular division remains unclear. Similarly, whether V^6^b+c is always located between the superior (S^6^) and basal (right S^7-10^ or left S^8-10^) divisions of the lung also remains unclear. In addition, bleeds often arise when using electrocautery to separate the intersegmental pulmonary parenchyma, owing to a damaged migratory pulmonary vein that passes through an adjoining lung segment, to a targeted lung segment. However, the division from which blood flows into the intersegmental veins remains undetermined, even though it is an important factor for separating lung parenchyma during segmentectomy.

Here we aimed to acquire detailed anatomic knowledge of the location and blood flow of the peripheral pulmonary veins V^3^a+b and V^6^b+c using novel volume-rendered 3D reconstruction of the pulmonary artery/vein and bronchus on CT images. Our results revealed critical information that can improve the outcomes of any type of segmentectomy.

## Methods

### Patient Population

We initially collected information on 135 consecutive patients who were treated by pulmonary resection, including lobectomy and segmentectomy, at Hiroshima University Hospital between January and December 2020. Among these were 22 patients with complete Digital Imaging and Communications in Medicine (DICOM) preoperative contrast-enhanced chest CT data. The Institutional Review Boards at the participating institutions approved this retrospective review of a prospective database and waived the requirement for informed consent from individual patients (E-2380; March 04, 2021).

### Volume-Rendering 3D Reconstruction of Pulmonary Artery/Vein and Bronchus

High-resolution images of chest tumors were acquired using a 16-row multidetector CT with the following parameters: 120 kVp, 70 keV, and 200 mA; section thickness, 1–2 mm; pixel resolution, 512 × 512; scan duration, 0.5–1.0 seconds; high spatial reconstruction algorithm with a 20-cm field of view and mediastinal (40 Hounsfield units [HU]; width, 400 HU) and lung (-600 HU; width, 1600 HU) window settings. All patients were evaluated by preoperative CT. Three-dimensional (3D) CT images were reconstructed to evaluate the anatomy of the pulmonary vessels and bronchi around V^3^a+b or V^6^b+c and to identify the dominant pulmonary arteries and veins in the target segment using REVORAS image processing software (Ziosoft). In brief, the pulmonary arteries and veins, bronchus, and each lobe of the lung were automatically extracted and drawn based on DICOM data of high-resolution CT using REVORAS, which accurately recognized the arteries and veins (Video 1).^13^ Thereafter, segmentectomy of the lingular division (S^4^ + S^5^) was simulated by designating the B^4^ and B^5^ bronchi as resected to evaluate the location, course, and peripheral blood flow of the pulmonary vein exposed in the intersegmental plane. The segment was then determined according to the bronchial dominant region. Segmentectomy of the upper division (S^1+2^ + S^3^) was similarly simulated by designating the bronchus of B^1+2^ and B^3^ as resected. The location, course, and peripheral blood flow of the intersegmental vein, bilateral V^6^b+c, were evaluated in the same way as the superior segment (S^6^).

## Results

### Clinicopathologic Findings of 22 Patients

Table 1 summarizes the characteristics of the study patients. Their median age was 70 years (range, 16 to 83 years), and 7 (32%) were female. The median Brinkman Index value was 745 (range, 0-2400). Height and body weight were 165 cm (range [IQR], 146.5-178 cm) and 58.3 kg (IQR, 46-73.5 kg), respectively. The postoperative diagnoses were 15 (68%) non–small cell lung cancers, 3 (14%) metastatic tumors, and 4 (18%) others. A hybrid video-assisted approach was used for all patients who were treated with thoracic surgery. The surgical procedures included 13 lobectomies (59%) and 9 segmentectomies (41%).

**Table 1:** Patient characteristics (N = 22)

Characteristic	Value
Age	70 (16-83)
Sex	
Female	7 (32)
Male	15 (68)
Brinkman Index, median (range)	745 (0-2400)
Height, cm, median (range)	165 (146.5-178)
Body weight, kg, median (range)	58.3 (46-73.5)
Diagnosis, n (%)	
Non–small cell lung cancer	15 (68)
Metastasis	3 (14)
Others	4 (18)
Approach, n (%)	
Open	0 (0)
Hybrid video-assisted thoracic surgery	22 (100)
Surgical procedure, n (%)	
Lobectomy	13 (59)
Segmentectomy	9 (41)

### Location and Peripheral Blood Flow of V^3^a+b, Intersegmental Pulmonary Vein Between Upper and Lingular Divisions of the Left Lung

**Figure 1:**
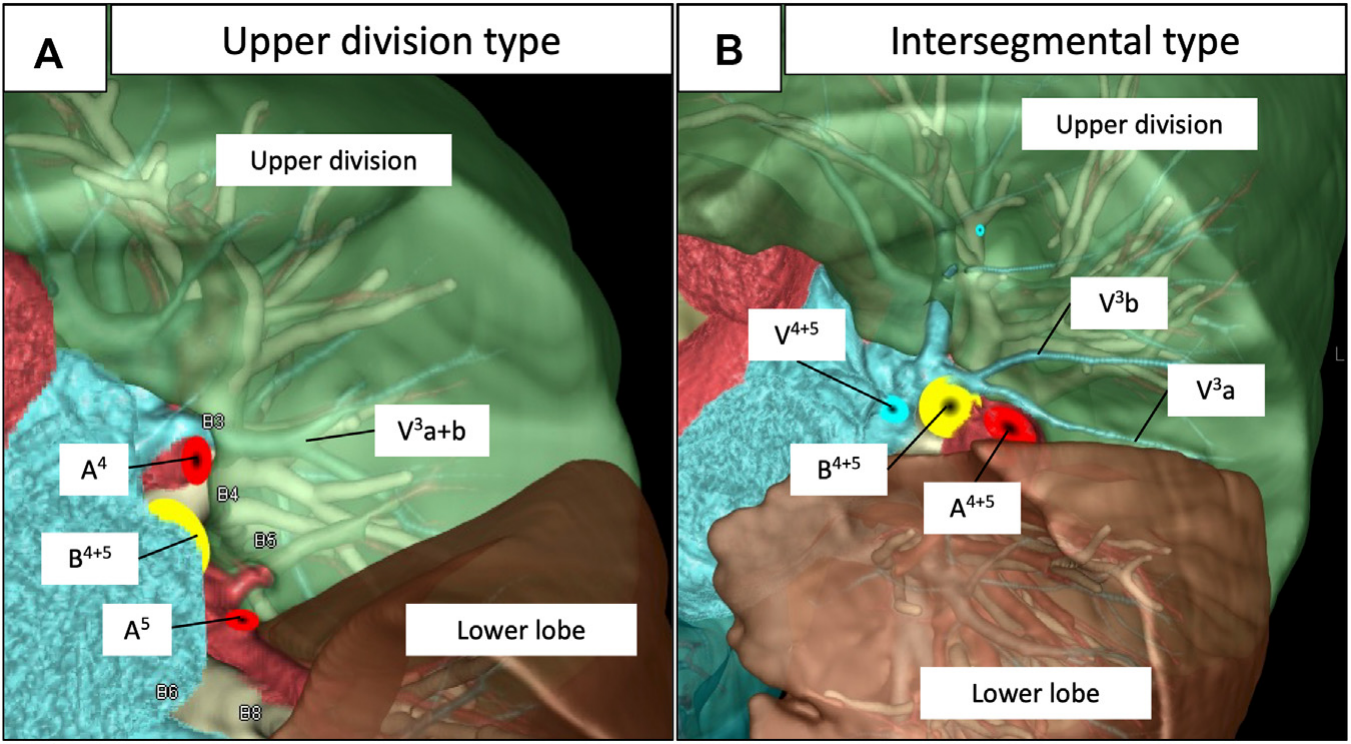
Representative images of V^3^a+b vein locations. A, Upper division type V^3^a+b veins located on the upper division. B, Intersegmental type V^3^a+b veins located on the intersegmental plane between the upper and lingular divisions of the left lung after simulated lingular segmentectomy (S^4+5^).

**Figure 2:**
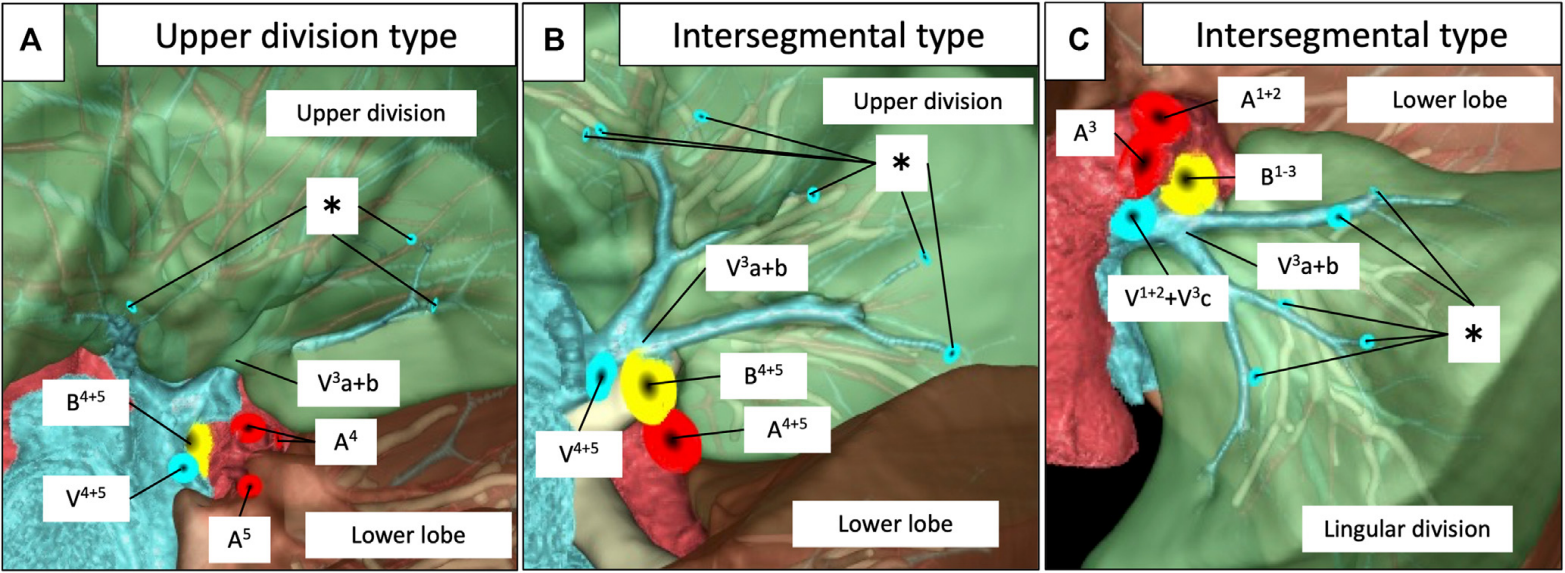
Representative images of peripheral pulmonary vein flow of V^3^a+b. A, Upper division type V^3^a+b from the lingular division (*∗*) after simulated lingular segmentectomy (S^4+5^). B, Intersegmental type V^3^a+b from the lingular division (*∗*) after simulated lingular segmentectomy (S^4+5^). C, Intersegmental type V^3^a+b from the upper division (*∗*) after simulated upper segmentectomy (S^1-3^).

Figure 1, *A* shows representative findings of the course of V^3^a+b, which was within the upper division, and Figure 1, *B* shows that of V^3^a+b on the intersegmental plane that was determined from the bronchial distribution. Eleven patients (50%) each had upper division and intersegmental types. The main root of V^3^a+b was not found in the lingular division (Table 2). Part of the V^3^a+b flow was derived from the lingular division in 14 patients (64%), including 4 with the upper division type and 10 with the intersegmental type (Figure 2 and Table 2). Figure 2, *B* and *C* shows that blood flowing in the intersegmental type of pulmonary veins was supplied by the lingular and upper divisions. Blood in all V^3^a+b veins partly flowed from the upper division regardless of type.

**Table 2:** Segments to which pulmonary veins belong and blood flow to intersegmental veins

Vein	Segment	Total (N = 22), n (%)	No flow from lingular/basal division, n (%)	Flow from lingular/basal division, n (%)
Left V3a+b	Upper division	11 (50)	7 (64)	4 (36)
	Intersegmental	11 (50)	1 (9)	10 (91)
	Lingular division	0 (0)	0 (0)	0 (0)
Right V6b+c	Superior division	13 (59)	12 (92)	1 (8)
	Intersegmental	6 (27)	1 (17)	5 (83)
	Basal division	0 (0)	0 (0)	0 (0)
	Undetermined	3 (14)	NA	NA
Left V6b+c	Superior division	10 (45)	8 (80)	2 (20)
	Intersegmental	10 (45)	4 (40)	6 (60)
	Basal division	0 (0)	0 (0)	0 (0)
	Undetermined	2 (9)	NA	NA

*NA*, Not applicable.

### Location and Peripheral Blood Flow of Intersegmental Pulmonary Vein V^6^b+c Between Superior and Basal Lung Divisions

**Figure 3:**
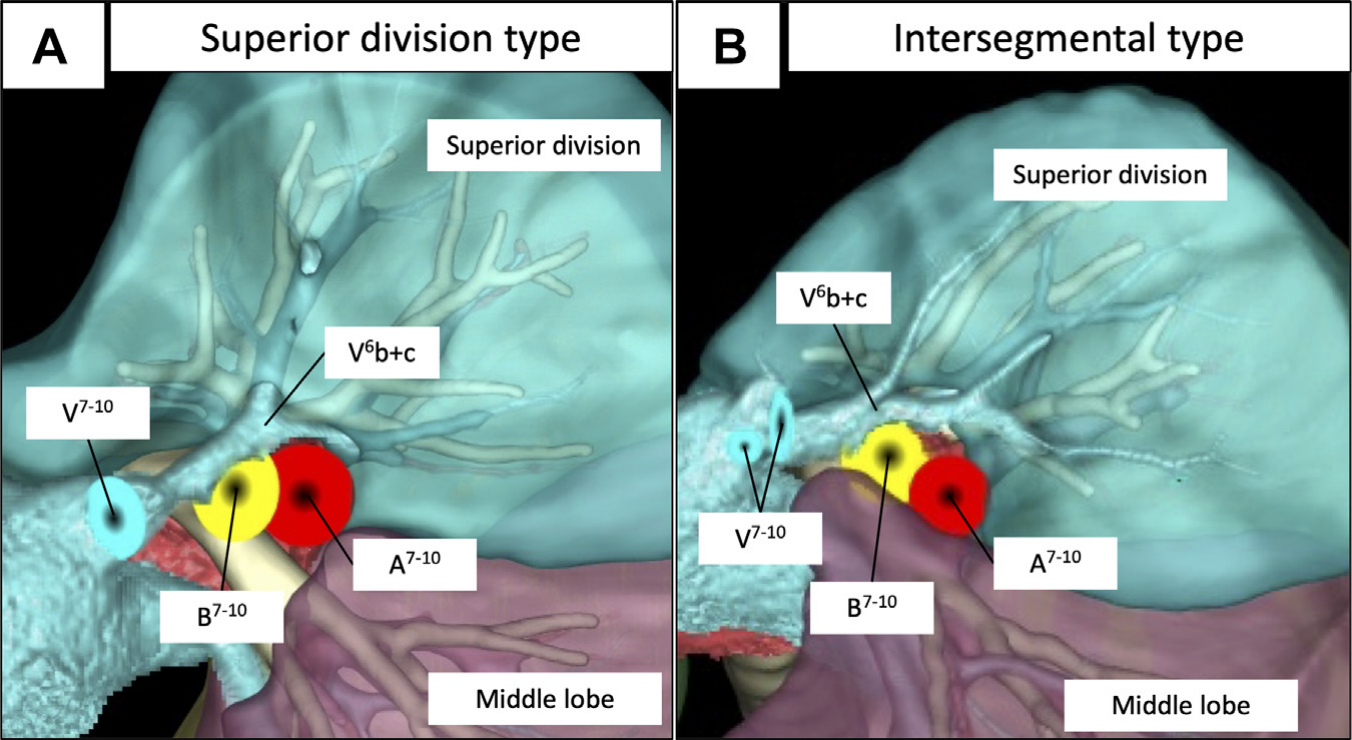
Representative images of right V^6^b+c vein locations. A, Superior division type V^6^b+c located on the superior division. B, V^6^b+c located on the intersegmental plane between the superior and basal divisions of the right lower lung lobe after simulated basal segmentectomy (S^7-10^).

**Figure 4:**
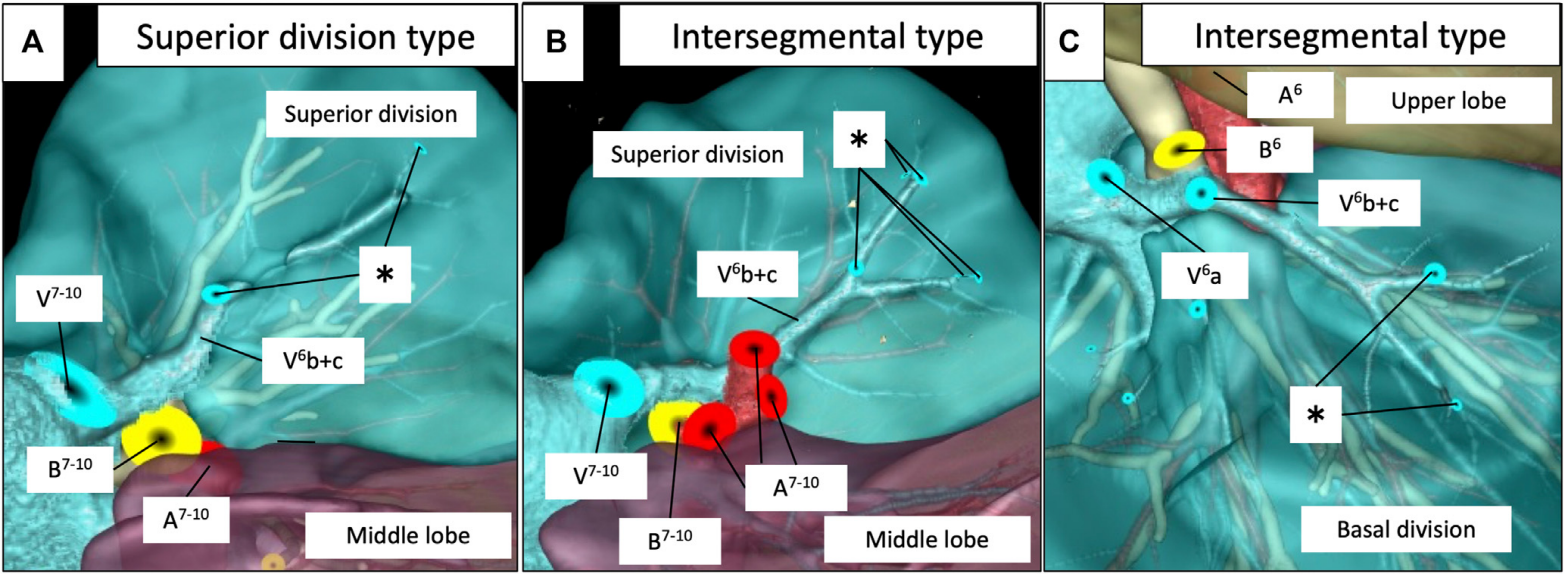
Representative images of blood flow in peripheral pulmonary veins of the right V^6^b+c vein. A, Superior division type V^6^b+c from the basal division (∗) after simulated basal segmentectomy (S^7-10^). B, Intersegmental type V^6^b+c from the basal division (*∗*) after simulated basal segmentectomy (S^7-10^). C, Intersegmental type V^6^b+c from the superior division (*∗*) after simulated superior segmentectomy (S^6^).

Figure 3, *A* shows representative findings of the course of the right V^6^b+c vein within the superior division, and Figure 3, *B* shows that the right V^6^b+c vein runs on the intersegmental plane determined by the bronchial distribution. Thirteen patients (59%) had the superior division type and 6 (27%) had the intersegmental type on the right lower lobe of the lung, and 10 patients (45%) each had these types on the left lower lobe. The distribution of the superior divisions was undetermined in the left lower lobe in 2 patients and the right lower lobe in 3 patients owing to a history of ipsilateral pulmonary surgery, tumor location, and a complicated bronchial distribution. The main root of V^6^b+c was not found in the basal division (Table 2). Part of the flow in the right and left peripheral pulmonary V^6^b+c veins was derived from the basal division in 6 patients (27%) and 8 patients (36%), respectively. Among these patients, 1 had had the upper division type in the right lower lobe, 2 had the upper division type left lower lobe, 5 had the intersegmental type in the right lower lobe, and 6 had the intersegmental type in the left lower lobe (Figure 4 and Table 2). Figure 4, *B* and *C* shows the blood flow of the intersegmental type of pulmonary veins supplied from the basal and superior divisions. Blood flowed from the superior division in all intersegmental V^6^b+c veins regardless of type.

Overall, patients 2, 10, 17, and 19 had only the upper/superior types of all 3 intersegmental pulmonary veins and no patients had only the intersegmental type (Table 3).

**Table 3:** Types of intersegmental pulmonary veins in the patients

Patient	V^3^a+b	Right V^6^b+c	Left V^6^b+c
1	Intersegmental	Intersegmental	Superior
2	Upper	Superior	Superior
3	Upper	Superior	Undetermined
4	Upper	Intersegmental	Superior
5	Intersegmental	Undetermined	Intersegmental
6	Upper	Intersegmental	Undetermined
7	Upper	Superior	Intersegmental
8	Intersegmental	Undetermined	Intersegmental
9	Intersegmental	Undetermined	Superior
10	Upper	Superior	Superior
11	Intersegmental	Superior	Superior
12	Upper	Intersegmental	Intersegmental
13	Intersegmental	Superior	Intersegmental
14	Intersegmental	Superior	Intersegmental
15	Intersegmental	Superior	Superior
16	Upper	Intersegmental	Intersegmental
17	Upper	Superior	Superior
18	Upper	Intersegmental	Intersegmental
19	Upper	Superior	Superior
20	Intersegmental	Superior	Intersegmental
21	Intersegmental	Superior	Superior
22	Intersegmental	Superior	Intersegmental

## Discussion

**Figure 5:**
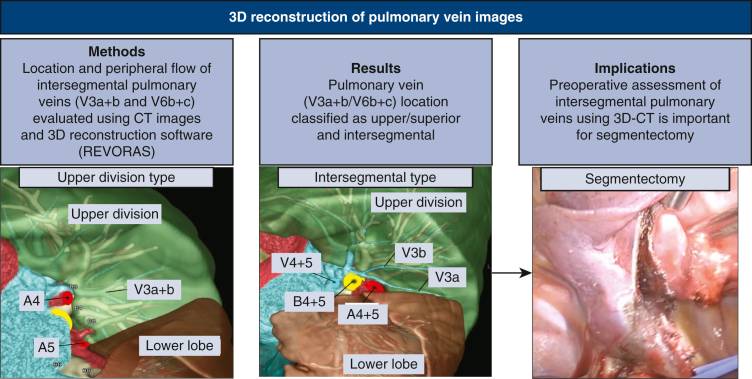
Preoperative evaluation of intersegmental pulmonary veins for segmentectomy with volume-rendering 3-dimensional computed tomography (*3D-CT*) image reconstruction.

This is the first study to reveal the detailed location, course, and peripheral blood flow in representative V^3^a+b and V^6^b+c intersegmental pulmonary veins, which are key structures in segmentectomy. The main root of the V^3^a+b vein in all patients was in the upper division or on the intersegmental plane between the upper and lingular divisions of the left lung, but not in the lingular division. Similarly, the main root of the V^6^b+c vein in all patients was found in the superior division or on the intersegmental plane between the superior and basal divisions of the right and left lower lobes of the lung, but never in the basal division. This anatomic information about the representative intersegmental left V^3^a+b and right/left V^6^b+c veins is valuable and meaningful for improving the outcomes of segmentectomy (Figure 5). A precise preoperative understanding of the bronchus and pulmonary vessels, especially the pulmonary veins, and preoperative simulation of segmentectomy will help avoid cutting misidentified blood vessels or the bronchus during challenging intraoperative identification.

The location and courses of intersegmental pulmonary veins were precisely assessed using novel volume-rendering 3D reconstruction of preoperative CT images. From an anatomic standpoint, the upper or superior type of intersegmental V^3^a+b or V^6^b+c veins do not need to be cut for segmentectomy of the lingular or basal divisions. Conversely, the upper or superior type of intersegmental V^3^a+b or V^6^b+c must be cut for segmentectomy of the upper or superior divisions, but the intersegmental pulmonary parenchyma should be separated along with V^3^a+b or V^6^b+c in patients with intersegmental type veins. The intersegmental type veins should be preserved during separation of the intersegmental parenchyma for upper or superior segmentectomy, to drain a remnant lung when a tumor is located far from the intersegmental plane. On the other hand, the intersegmental veins should be cut to stop drainage from a tumor when it is large or located near the intersegmental plane. If the intersegmental pulmonary vein is preserved, surgeons must carefully attend to bleeding while separating the intersegmental pulmonary parenchyma in a patient with intersegmental veins compared with upper or superior type veins. In terms of basal or lingular segmentectomy, the intersegmental type of pulmonary veins should be preserved. Under such circumstances, preoperative 3D imaging can predict a greater likelihood of a bleed arising from the intersegmental type of veins compared with the upper or superior type during intersegmental separation. Of course, the line of pulmonary parenchymal separation will need to change from the anatomic intersegmental line when a tumor is located nearby to secure a surgical margin. Nevertheless, preoperative information from 3D volume-rendering image reconstruction is valuable for determining whether V^3^a+b or V^6^b+c veins should be cut for upper or superior segmentectomy.

In terms of the migratory peripheral pulmonary vein that passes through a nearby lung segment, to a targeted lung segment according to 3D volume-rendering image reconstruction, some blood occasionally flows from the peripheral pulmonary vein of adjoining segments into intersegmental veins from the lingular and basal divisions for V^3^a+b and V^6^b+c, respectively. Flow from the lingular/basal division was more frequent in the intersegmental type than in the upper/superior type in the left upper/bilateral lower lobe. Preoperatively understanding peripheral vein flow with 3D volume-rendering image reconstruction can allow easier separation of the intersegmental pulmonary parenchyma. Appropriate stapling might be advisable rather than electrocautery to control copious blood flow from an adjoining segment. In addition, even if pulmonary parenchyma is separated using electrocautery, the bleeding from the migratory peripheral pulmonary veins that can be anticipated with preoperative 3D imaging is easy to manage. Thus, surgeons can select electrocautery or staples according to their preference.

We determined the segment based on bronchial distribution, but it also could be determined based on the pulmonary artery. We applied the method based on the bronchus because it varies less frequently than the pulmonary artery.^14^ Inflation–deflation has been compared with infrared indocyanine green staining to identify the intersegmental plane, and the 2 methods were found to be completely concordant in small patient cohorts.^15^ However, whether the distribution of the bronchus is always consistent with that of the pulmonary artery remains unknown. Defining differences between segments based on the pulmonary artery and the bronchus will be the next target of interest.

Because types of veins differ among intersegments even in the same patient, precise preoperative evaluation using 3D volume-rendering image reconstruction of the location and courses of the intersegmental pulmonary veins in each intersegment is important before starting any type of segmentectomy. In contrast, images of superior segments could not be reconstructed in all cases. One reason for this was a centrally located tumor near the root of the lower bronchus; another is that the patient had undergone 2 surgical pulmonary resections of the same lower lobe. Others could not be determined because of the complexity of the bronchial branch distribution within the lower lobe. This is a limitation of 3D CT image reconstruction. However, evaluating the pulmonary arteries, veins, and bronchus in such circumstances using alternative modalities is also difficult.

This study has some limitations. Evidence is weaker in retrospective studies than in prospective studies. Many more pulmonary vein samples are needed to validate our results from a small patient cohort. In addition, location patterns on the intersegmental pulmonary veins should be validated for patients who will be treated by segmentectomy. Despite these limitations, however, our findings provide important and novel anatomic insights for any type of segmentectomy.

In conclusion, precise evaluation of the intersegmental veins, such as V^3^a+b and V^6^b+c, using preoperative volume-rendering 3D reconstruction of CT images can provide useful anatomic information to aid safe and reliable separation of the intersegmental pulmonary parenchyma.

### Conflict of Interest Statement

The authors reported no conflicts of interest.

The *Journal* policy requires editors and reviewers to disclose conflicts of interest and to decline handling or reviewing manuscripts for which they may have a conflict of interest. The editors and reviewers of this article have no conflicts of interest.

## Supplementary Data

Video 1 Summary of preoperative evaluation of intersegmental pulmonary veins with volume-rendering 3-dimensional computed tomography image reconstruction. Video available at: https://www.jtcvs.org/article/S2666-2507(22)00464-3/fulltext.
