# Supplementary figures and images for: The intersegmental pulmonary vein is not always located on the intersegmental plane of the lung: Evaluation with 3-dimensional volume-rendering image reconstruction
Source: JTCVS Tech. 2022 Sep 13;16:132–8. doi: 10.1016/j.xjtc.2022.09.002 (PMC9735388; doi:10.1016/j.xjtc.2022.09.002)

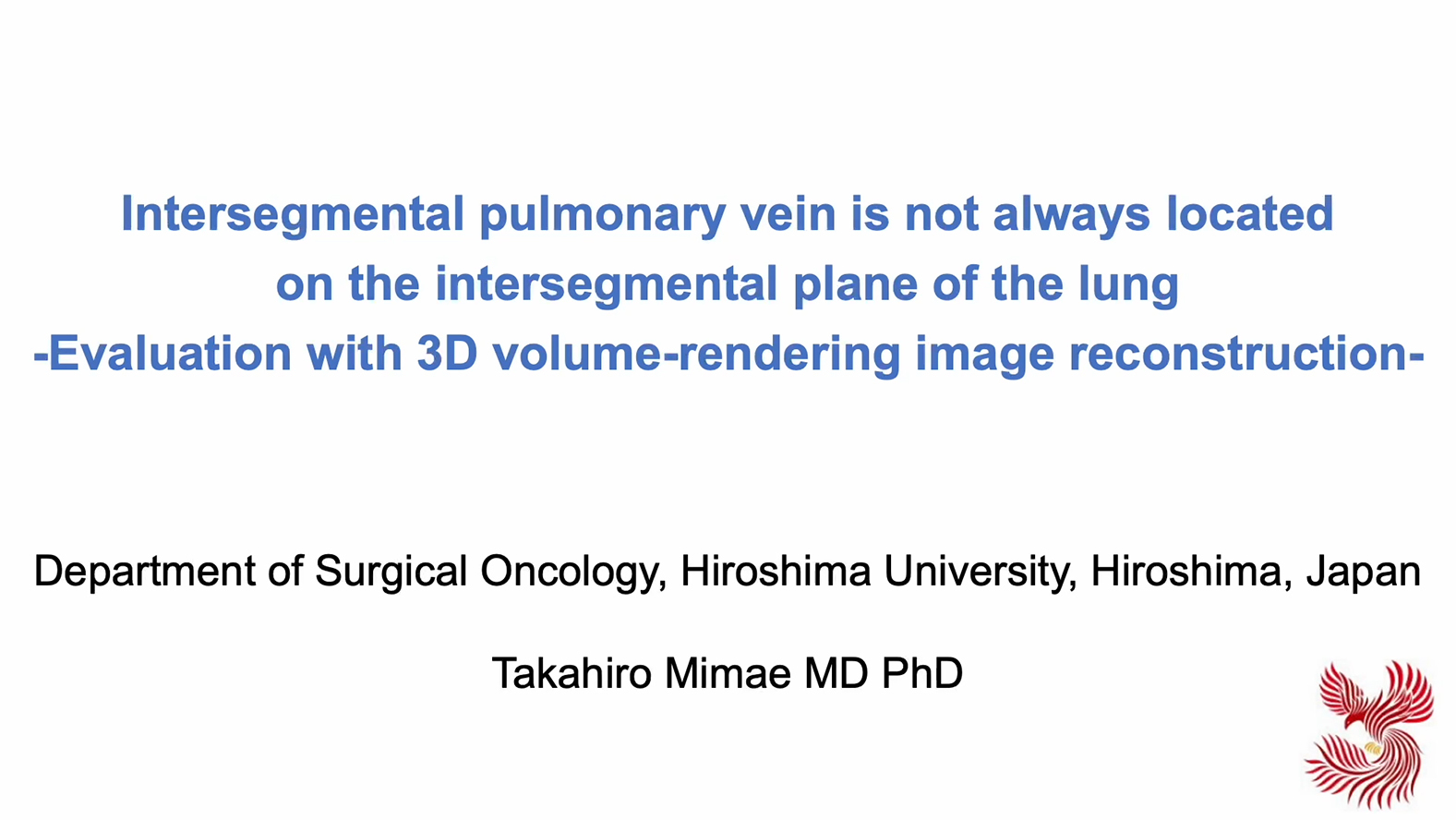

Supplement: Video 1 — Summary of preoperative evaluation of intersegmental pulmonary veins with volume-rendering 3-dimensional computed tomography image reconstruction. Video available at: https://www.jtcvs.org/article/S2666-2507(22)00464-3/fulltext. [file fx3.jpg]
